# N^6^-Methyladenosine (m^6^A)-Mediated Regulation of Lipid Metabolism: Molecular Mechanisms, Pathological Implications, and Therapeutic Perspectives

**DOI:** 10.3390/biom16010101

**Published:** 2026-01-07

**Authors:** Qingjun Zhu, Yunyi Hu, Minhao Li, Haili Yang, Le Zhao, Yongju Zhao

**Affiliations:** 1 College of Animal Science and Technology, Southwest University, Chongqing 400715, China; zqj1120@email.swu.edu.cn (Q.Z.); hyy231717@email.swu.edu.cn (Y.H.); lmh112022328001897@email.swu.edu.cn (M.L.); yhl2017@swu.edu.cn (H.Y.); 2 Chongqing Key Laboratory of Herbivore Science, Chongqing 400715, China

**Keywords:** m^6^A modification, lipid metabolism, metabolic diseases, inter-organ crosstalk, therapeutic targets

## Abstract

Dysregulated lipid metabolism constitutes the fundamental etiology underlying the global burden of obesity and its associated metabolic disorders. N^6^-methyladenosine (m^6^A) is the most abundant reversible chemical modification on messenger RNA and influences virtually every aspect of RNA metabolism. Recent studies demonstrate that m^6^A mediates regulatory networks governing lipid metabolism and contributes to the pathogenesis of multiple metabolic diseases. However, the precise roles of m^6^A in lipid metabolism and related metabolic disorders remain incompletely understood. This review positions m^6^A modification as a central epigenetic switch that governs lipid homeostasis. We first summarize the molecular components of the dynamic m^6^A regulatory machinery and delineate the mechanisms by which it controls key lipid metabolic processes, with an emphasis on adipogenesis, thermogenesis and lipolysis. Building on this, we further discuss how dysregulated m^6^A acts as a shared upstream driver linking obesity, type 2 diabetes (T2D), metabolic dysfunction-associated steatotic liver disease (MASLD), and insulin resistance through tissue-specific and inter-organ communication mechanisms. We also evaluate the potential of targeting m^6^A regulators as therapeutic strategies for precision intervention in metabolic diseases. Ultimately, deciphering the complex interplay between m^6^A modification and lipid homeostasis offers a promising frontier for the development of epitranscriptome-targeted precision medicine against obesity and its associated metabolic disorders.

## 1. Introduction

Dysregulated lipid metabolism drives major metabolic disorders, with obesity and related pathologies—such as type 2 diabetes (T2D), metabolic dysfunction-associated steatotic liver disease (MASLD), and cardiovascular disease—representing a primary global health challenge [1,2,3]. As adipose tissue (AT) functions as a vital energy reservoir and endocrine signaling hub, its normal development, differentiation, and functionality are essential for the maintenance of system-wide metabolic homeostasis [4,5,6,7]. Therefore, a detailed understanding of the molecular machinery governing lipid metabolism is essential for developing effective disease prevention strategies and therapeutics.

Traditional research on metabolic diseases has largely focused on genetic predispositions and environmental influences, which exert their effects primarily through classical epigenetic mechanisms such as DNA methylation and histone modifications [8]. However, the discovery of the epitranscriptome—representing a dynamic and reversible regulatory layer at the RNA level—has heralded a new era in molecular biology [9,10]. N^6^-methyladenosine (m^6^A) is by far the most abundant and best-characterized internal eukaryotic mRNA modification [11]. This modification is not an indelible tag; rather, it is dynamically “written” by methyltransferases (e.g., METTL3, METTL14), “erased” by demethylases (e.g., FTO, ALKBH5), and recognized by a suite of binding proteins (e.g., YTHDF1-3, YTHDC1-2, IGF2BP1-3). This interplay determines the fate of target transcripts by modifying splicing, nuclear export, stability, and translation efficiency [12]. The seminal discovery that the fat mass and obesity-associated (*FTO*) gene encodes an m^6^A demethylase provided the first direct link between epitranscriptomics and the pathophysiology of energy metabolism and obesity, igniting immense interest in the field [13]. More recent research has revealed that m^6^A modification is deeply involved in numerous processes such as lipolysis, thermoregulation, and adipocyte differentiation. Furthermore, it is intricately linked to the pathogenesis of obesity as well as other metabolic diseases such as steatotic liver disease [14,15,16]. Crucially, this regulatory system functions as a pivotal “metabolic rheostat” capable of sensing cellular energy status and modulating metabolic responses. By controlling the fate of key metabolic gene transcripts, it reprograms cellular metabolic pathways, thereby establishing a sophisticated feedback loop [14,17].

This review aims to comprehensively explore the pleiotropic roles of m^6^A RNA modification in lipid metabolism and related metabolic disorders. We first summarize the molecular components and dynamic regulatory mechanisms of the m^6^A machinery, followed by an in-depth analysis of its regulatory networks in key metabolic processes, including adipogenesis, AT browning, and thermogenesis. On this basis, we further discuss how the dysregulation of m^6^A acts as a convergent pathophysiological mechanism driving the development of obesity, MASLD, and insulin resistance. Finally, we discuss the therapeutic potential of targeting m^6^A regulators, along with current challenges in both research and clinical applications.

## 2. Molecular Mechanism of m^6^A Modification

**Figure 1:**
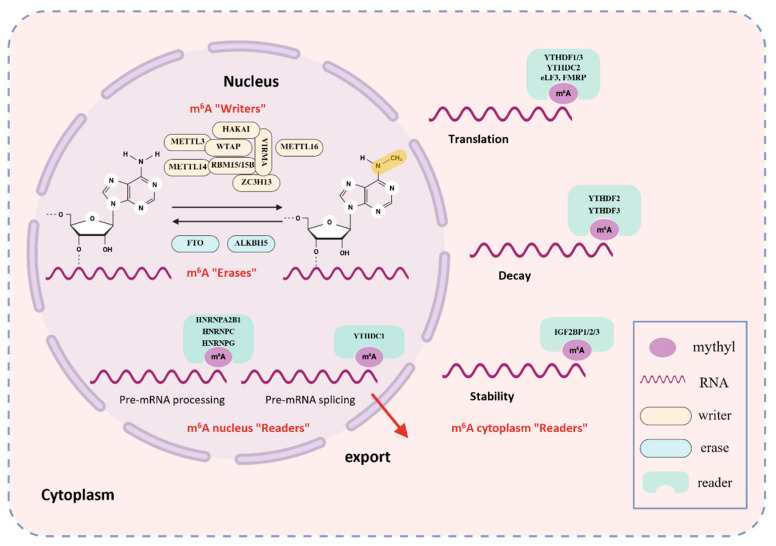
The Dynamic Modification Mechanism of m^6^A. m^6^A methylation is catalyzed by a multi-protein “writer” complex, primarily composed of METTL3, METTL14, WTAP, RBM15, VIRMA, and ZC3H13. METTL16 can also install m^6^A marks in specific contexts. These marks are removed by “erasers”, chiefly the demethylases FTO and ALKBH5. m^6^A sites on RNA are recognized by diverse “reader” proteins-including YTHDF1/2/3, YTHDC1/2, HNRNPA2B1/C, and IGF2BP1/2/3—which bind methylated transcripts and modulate their fate by affecting splicing, stability, translation, and degradation.

The m^6^A modification is predominantly found within a conserved consensus sequence, RRACH (where R = G or A; H = A, C, or U), and is enriched near stop codons and in 3′ untranslated region (3′-UTR) of mRNA transcripts [18]. Its biological functions are mediated by a sophisticated molecular network comprising three key classes of proteins: “writers,” “erasers,” and “readers.” These three components work synergistically to dynamically regulate m^6^A modification, thereby influencing mRNA stability [19,20], splicing [21], translation [22,23], and degradation [24]. Consequently, m^6^A modification regulates the RNA fate, thereby affecting cellular functions and physiological processes [25] (As shown in Figure 1). While the majority of research has focused on mRNA, emerging evidence reveals that m^6^A modification also occurs on a wide array of other RNA species, including rRNA, snRNA, miRNA, lncRNA, and circRNA [26]. This broad involvement indicates that m^6^A plays a widespread role in cellular regulation, including the control of intricate metabolic networks. These findings open new perspectives for understanding how m^6^A contributes to metabolic homeostasis and the onset and disease progression.

### 2.1. m^6^A Writers

The deposition of m^6^A is catalyzed by a multi-subunit methyltransferase complex (MTC) that is predominantly located in the nucleus. The catalytic core of this complex is a stable heterodimer composed of the core subunits Methyltransferase-like 3 (METTL3) and Methyltransferase-like 14 (METTL14). METTL3 serves as the primary catalytic subunit responsible for transferring methyl groups from S-adenosylmethionine, while METTL14 functions as an RNA-binding scaffold that enhances the complex’s affinity for substrate RNA and activates METTL3 via allosteric regulation [27]. Additionally, m^6^A METTL-associated complexes (MACOMs) involved in localization and stabilization—such as WTAP [28], VIRMA [29], ZC3H13 [30], HAKAI [31], and RBM15/15B [32]—coordinate methylation within RRACH sequences. Among these, Wilms’ tumor 1-associating protein (WTAP) plays a critical role in directing the complex to nuclear speckles, which serve as RNA processing centers [28]. Vir-like m^6^A methyltransferase associated protein (VIRMA) guides preferential m^6^A methylation at the 3′UTR and near stop codons [29]. Zinc finger CCCH domain-containing protein 13 (ZC3H13) stabilizes the interaction between WTAP and RBM15 and helps to anchor the writer complex within the nucleus [30,33]. E3 ubiquitin-protein ligase (HAKAI) is critical for maintaining MTC stability, although its precise mechanistic role in m^6^A deposition requires further investigation [31]. The RNA-binding proteins RBM15 and its paralog RBM15B facilitate substrate recognition; they bind to U-rich sequences on mRNA and recruit the writer complex to nearby adenosine residues for methylation [32]. Moreover, Methyltransferase-like 16 (METTL16) has been shown to catalyze methylation at the UACAGAGAA motif within a specific hairpin structure in the MAT2A 3′UTR [34].

### 2.2. m^6^A Erasers

The reversibility of m^6^A modification is ensured by two characterized demethylases, the FTO and AlkB homolog 5 (ALKBH5). FTO utilizes iron ions and 2-oxoglutarate (α-KG) as cofactors for its catalytic function. Through stepwise oxidation reactions, it first converts the methyl group in m^6^A into hydroxymethyladenosine (hm^6^A) and then, through oxidation, converts it into a formyl ester and thus brings about demethylation [35]. Importantly, FTO maintains broad substrate availability, targeting not only m^6^A but also other modifications such as N^6^,2′-O-dimethyladenosine (m^6^Am) and N^1^-methyladenosine (m^1^A) [35]. ALKBH5 exhibits catalytic activity similar to that of FTO, utilizing iron ions and α-KG to remove m^6^A modifications through oxidation. However, ALKBH5 is more specific, recognizing m^6^A solely on single-stranded RNA, and exhibits cell-type–dependent activity [36]. Despite both functioning as m^6^A demethylases, they are distinct in terms of substrate preference and cellular function. FTO acts more universally across various RNA modifications (m^6^A, m^6^Am, m^1^A), whereas ALKBH5 exhibits strong specificity for m^6^A. In certain disease conditions, such as cancer, both enzymes may coordinately orchestrate RNA metabolism through synergistic or overlapping activities, thus influencing disease development [37].

### 2.3. m^6^A Readers

The functional effects of m^6^A are interpreted by a diverse group of “reader” proteins. Among these, the YT521-B homology (YTH) domain-containing family is the most extensively studied. These proteins directly recognize and bind to m^6^A-modified sites via a characteristic hydrophobic pocket within their YTH domain, which specifically accommodates the methylated adenosine [38]. Research indicates that YTHDF1 promotes the translation efficiency of m^6^A-modified mRNAs by binding to m^6^A modification sites and recruiting translation initiation factors such as eIF3. This process accelerates mRNA transport onto ribosomes, enabling m^6^A-labeled mRNAs to be prioritized for translation [24]. As a primary inducer of mRNA degradation, YTHDF2 recognizes m^6^A sites through its YTH domain, subsequently recruiting the CCR4-NOT deadenylase complex to promote deadenylation and degradation of target mRNAs [24]. This mechanism is crucial for rapid cellular clearance of unnecessary mRNAs and dynamic regulation of gene expression programs. YTHDF3 exhibits more complex functions and is considered a synergistic partner of both YTHDF1 and YTHDF2. It can co-activate translation with YTHDF1 and synergistically promote mRNA degradation with YTHDF2, potentially acting as a regulator to accelerate the overall metabolic turnover of m^6^A-modified mRNAs [39]. In the nucleus, YTHDC1 is the primary m^6^A reader and has multiple functions. It regulates the alternative splicing of pre-mRNAs by recruiting or repelling splicing factors like SRSF3 and SRSF10 [40]. Additionally, YTHDC1 facilitates the nuclear export of mature m^6^A-modified mRNAs by interacting with export factors such as NXF1 [9]. Members of the heterogeneous nuclear ribonucleoprotein (HNRNP) family, such as HNRNPA2B1 and HNRNPC, also recognize m^6^A sites in the nucleus. They regulate splicing and influence RNA secondary structure, thereby shaping subsequent RNA metabolic processes [41,42]. YTHDC2, another cytoplasmic YTH-domain protein, enhances translation efficiency by recognizing m^6^A marks within the coding sequence. Unlike other YTH proteins, it exhibits a lower binding affinity for m^6^A, suggesting it may bind to specific contexts or employ a distinct mode of action [43]. Furthermore, insulin-like growth factor 2 mRNA-binding proteins 1–3 (IGF2BP1–3) directly bind m^6^A RNA through their KH domains. In an m^6^A-dependent manner, they stabilize thousands of potential target mRNAs and enhance their translation, thereby broadly influencing gene expression outcomes [44]. Furthermore, other proteins such as G3BP1 and FMR1 have also been identified as m^6^A readers that respectively regulate the stability and translation rate of modified mRNAs [45].

### 2.4. m^6^A Modification in the Regulation of Adipocyte Development and Metabolism

Adipocytes play a central role in energy storage, metabolic regulation, and endocrine function. Their plasticity—the ability to expand, remodel, and switch functions in response to physiological demands—is essential for maintaining metabolic homeostasis [46,47]. FTO, a classical mRNA m^6^A demethylase, was the first gene identified to have the strongest genetic association with polygenic obesity and has been repeatedly linked to obesity in numerous studies [48,49]. The discovery of FTO as the first m^6^A mRNA demethylase established the concept of reversible RNA modification, laying the groundwork for integrating RNA methylation into adipose biology research [21]. Increasing evidence further indicates that m^6^A modification regulates the expression of transcription factors and AT–specific genes, thereby shaping lipid metabolic processes. Its role in adipocyte development and metabolic function merits deeper exploration and understanding.

### 2.5. Regulation of Adipogenesis by m^6^A Modification

**Figure 2:**
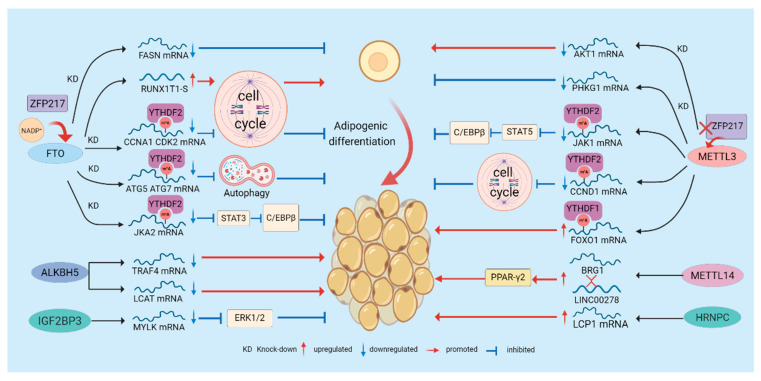
Regulation of Adipogenesis by m^6^A Modification. m^6^A regulates adipogenesis through multiple mechanisms. “writers”, “erasers”, and “readers” dynamically modulate m^6^A modification, thereby influencing the degradation and translation of adipogenic regulators and affecting pathways such as the cell cycle and autophagy to control adipocyte differentiation. Moreover, certain regulatory factors or metabolites, such as NADP^+^ and ZFP217, can alter m^6^A modification and further modulate adipogenesis.

Adipogenesis, the differentiation of pre-adipocytes into mature, lipid-storing adipocytes, is a highly orchestrated process governed by a cascade of transcription factors and signaling pathways [50]. m^6^A modification exerts a critical bidirectional regulatory role in this process, highlighting the importance of maintaining balanced m^6^A levels in determining cell fate. Numerous functional studies show that *FTO* overexpression promotes adipogenesis by globally reducing cellular m^6^A levels, whereas *FTO* knockdown (KD) inhibits differentiation [51] (As shown in the left panel of Figure 2). Mechanistically, FTO modulates m^6^A marks near splice junctions to alter alternative splicing of the adipogenic regulator RUNX1T1, promoting production of a pro-adipogenic short isoform that drives mitotic clonal expansion(MCE)—a key early event in adipogenic commitment [52,53]. FTO also sustains cell-cycle progression by preserving expression of key regulators (e.g., *CCNA2*, *CDK2*). By preventing m^6^A accumulation on these transcripts, FTO averts YTHDF2-mediated decay and thereby supports cell-cycle progression and clonal expansion. Conversely, *FTO* KD increases m^6^A on these mRNAs, promoting YTHDF2-dependent degradation, prolonging the cell cycle, and inhibiting adipogenesis [54]. Autophagy is another FTO-regulated pathway: *FTO* deficiency elevates m^6^A on *ATG5* and *ATG7* mRNAs, triggering YTHDF2-dependent degradation, which suppresses essential autophagic activity and impairs adipocyte maturation [55]. Additionally, FTO modulates transcripts involved in lipid biosynthesis (e.g., *FASN*) and signaling pathways such as JAK2–STAT3–C/EBPβ; by altering m^6^A levels on these mRNAs, FTO affects their stability and expression to promote an adipogenic phenotype [56,57]. In contrast to FTO’s pro-adipogenic role, METTL3 acts as an adipogenesis suppressor in multiple models (As shown in the right panel of Figure 2). METTL3 cooperates with YTHDF1 to increase m^6^A on *FOXO1* mRNA, enhancing its transcriptional activity and thereby inhibiting adipocyte differentiation [58]. In a model of *pig* bone marrow mesenchymal stem cells differentiating into adipocytes, METTL3 targets and inhibits the JAK1/STAT5/C/EBPβ signaling pathway through an m^6^A-YTHDF2-dependent mechanism, suppressing BMSC adipocyte differentiation [59]. This contrasts sharply with the positive regulation of a related pathway (JAK2–STAT3–C/EBPβ) by FTO, underscoring the finely tuned balance within the m^6^A regulatory system. More recent studies have shown that *METTL3* KD reduces m^6^A modification of *PHKG1* mRNA, leading to decreased *PHKG1* expression. This reduction, in turn, upregulates downstream adipogenic genes and promotes adipocyte differentiation. In addition, loss of *METTL3* decreases *AKT1* expression in an m^6^A-dependent manner, which further favors adipogenic differentiation [60,61]. These discrepancies indicate that METTL3′s functional outcomes depend strongly on cell identity, the repertoire of target transcripts, and developmental or stimulatory context, underscoring the complex regulatory roles of m^6^A writers within adipogenic networks. Other m^6^A regulators also participate in adipogenesis. METTL14 modulates m^6^A on the long noncoding RNA *LINC00278*, enhancing its interaction with the chromatin remodeler BRG1 and activating the PPARγ2 pathway to promote adipogenesis, suggesting that writers can regulate adipogenesis via lncRNA-mediated epigenetic/chromatin mechanisms [62]. The demethylase ALKBH5 influences adipogenesis by modulating the stability of transcripts such as *LCAT* and *TRAF4*, indicating that distinct demethylases have specific roles in adipogenic regulation [63,64]. Reader proteins show diverse functions: YTHDC2 directly binds mRNAs of lipogenic genes (e.g., *SREBP1*, *FASN*, *SCD1*, *ACC*) and reduces their stability, thereby suppressing lipid biosynthesis [65]. HNRNPC binds an m^6^A motif in *LCP1* mRNA to enhance its stability and modulate the cytoskeleton in a manner that favors adipogenesis [66]. IGF2BP3, in an m^6^A-dependent manner, stabilizes *MYLK* mRNA and subsequently suppresses ERK1/2 phosphorylation, which impedes adipogenic differentiation [67]. Together, these findings indicate that reader families act as selective “amplifiers” or “suppressors” of adipogenic fate by binding distinct sets of transcripts.

The adipogenic m^6^A network is further regulated upstream by metabolites and transcription factors: NADP^+^ acts as an allosteric activator of FTO, directly enhancing its demethylase activity and promoting adipogenesis, underscoring how metabolic state feeds back to epitranscriptomic enzymes [14]. The zinc-finger protein ZFP217 coordinates FTO activity by activating FTO transcriptionally and preserving FTO function post-transcriptionally via interaction with YTHDF2, thereby amplifying demethylation and driving adipogenesis. Conversely, *ZFP217* loss upregulates *METTL3*, increases m^6^A on *CCND1*, promotes its degradation, and inhibits MCE and adipogenesis [68,69,70]. Finally, m^6^A is not confined to mRNA: METTL5-mediated m^6^A of 18S rRNA is critical for 80S ribosome assembly and selective translation. *METTL5* loss selectively reduces the translational efficiency of key fatty acid metabolism enzymes (e.g., ACSL4), thereby inhibiting adipogenesis and highlighting rRNA m^6^A as an additional regulatory layer in lipid metabolism [71].

### 2.6. Regulation of Thermogenesis by m^6^A Modification

Mammals possess two main types of functional AT: white adipose tissue (WAT), which stores energy, and brown adipose tissue (BAT), which expends energy to generate heat. BAT uses uncoupling protein 1 (UCP1) to convert chemical energy into heat, playing a critical role in maintaining body temperature and energy balance [72,73]. Additionally, under cold exposure or β3-agonist stimulation, WAT can transform into energy-expending beige adipose through a process called “browning,” offering a promising strategy to combat obesity. m^6^A modification acts as an energy switch, determining whether cells favor energy storage or expenditure. Genetic and molecular evidence directly implicates the m^6^A demethylase FTO in the regulation of thermogenesis. For example, functional variants at the FTO locus (e.g., rs1421085) have been shown to affect mitochondrial thermogenesis and browning in preadipocytes, suggesting a role for this locus in adipocyte fate determination [74]. Conversely, in animal and cellular models, *FTO* KD is typically associated with increased *UCP1* expression and enhanced WAT browning, whereas *FTO* overexpression suppresses thermogenic phenotypes and favors maintenance of white adipocyte characteristics [75,76]. Mechanistically, FTO modulates thermogenic programs by altering m^6^A levels on key transcripts: One mechanistic study showed that *FTO* KD increases m^6^A on hypoxia-inducible factor 1α (*HIF1A*) mRNA; the modified transcript is recognized by the nuclear reader YTHDC2, which enhances *HIF1A* translation. Elevated HIF1A then activates thermogenic programs such as PGC1α and PRDM16, upregulating UCP1 and driving WAT browning [77]. In porcine adipocytes, FTO-mediated demethylation of Plin5 mRNA increases Plin5 protein levels, reduces lipid droplet size, enhances triglyceride turnover and mitochondrial respiratory function, and promotes thermogenesis [78]. Additionally, FTO has been reported to regulate BAT metabolism and WAT browning via miRNA expression changes, although the precise regulatory mechanisms remain to be elucidated [79].

In contrast to FTO, m^6^A writers generally promote thermogenesis in multiple contexts. Adipose-specific knockout(KO) of METTL3 substantially reduces tissue m^6^A levels and impairs BAT maturation and function, leading to decreased *PRDM16*, *PPARγ*, and *UCP1* expression and compromised adaptive thermogenesis [80]. Concurrently, multiple studies demonstrate that METTL3 stabilizes or promotes translation of thermogenic transcripts (e.g., *KLF9*) and, together with readers such as IGF2BP2, maintains expression of glycolytic genes in beige adipocytes (*HK2*, *PFKL*, *PKM*), thereby supporting the substrate supply and energetic switch required for browning [81,82]. *METTL14* deficiency reduces m^6^A content on mRNA for β-adrenergic receptor genes (*ADRB2*, *ADRB3*) and lipolytic genes (*ATGL*, *CGI-58*), increasing their translation and protein abundance in adipocytes. This augments β-adrenergic signaling and lipolysis, ultimately impairing thermogenic capacity [15]. Moreover, WTAP, a component that stabilizes the METTL3 complex, has been shown to participate in BAT development and energy metabolism by modulating writer complex stability and activity, underscoring the importance of complex integrity for thermogenic function [83].

m^6^A writers and erasers generate the epitranscriptomic “signal,” while distinct reader proteins determine how that signal is functionally interpreted. In adipocytes, YTHDF1 promotes the m^6^A-dependent translation of BMP8B, thereby inducing WAT browning [84]. By contrast, reader proteins such as IGF2BP2 can selectively modulate the translation or stability of mitochondrial proteins, including UCP1. IGF2BP2-deficient mice display increased UCP1 protein levels, enhanced translation of mitochondrial proteins, and resistance to HFD-induced obesity. In vitro reporter assays containing the UCP1 untranslated regions show that IGF2BP2 suppresses reporter translation, suggesting that IGF2BP2 binds UCP1 UTRs to reduce translation efficiency [85]. Moreover, SIRT7 deacetylates IGF2BP2 and thereby strengthens its inhibitory effect on UCP1 mRNA translation, suppressing brown adipose thermogenesis and revealing an upstream regulatory mechanism of IGF2BP2-mediated translational control [86]. Intriguingly, the circular RNA circ_0001874 interacts with IGF2BP2 and UCP1 to increase *UCP1* translation and enhance thermogenesis [87]. Recent work also highlights the nuclear reader YTHDC1 as important for BAT development and *PPARγ* stability, revealing that readers can both regulate translation and influence protein homeostasis, including ubiquitination pathways [88]. Overall, a reader’s target specificity and functional domains determine whether an identical m^6^A mark is decoded to promote translation/stability or to trigger degradation/inhibit translation, thereby shaping thermogenic phenotypes.

**Figure 3:**
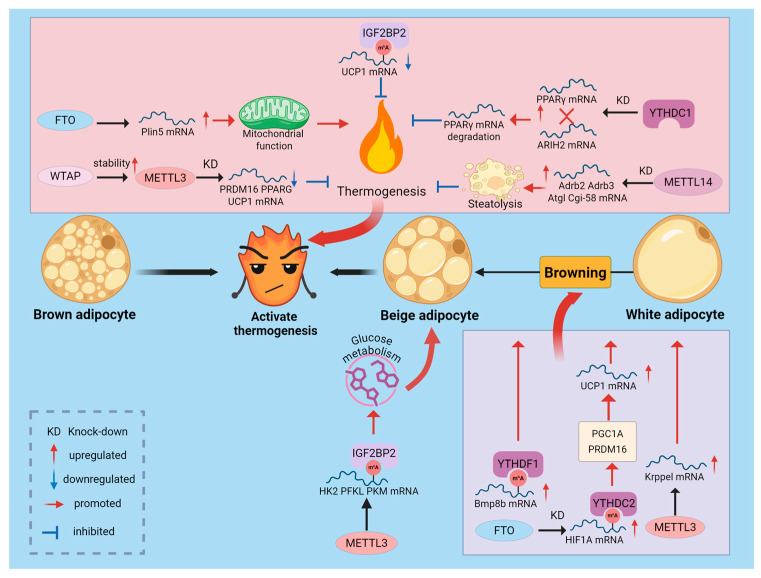
Regulation of Adipose Thermogenesis by m^6^A Modification. m^6^A modification acts as an energy switch in the thermogenic process. FTO and METTL3/METTL14, by modulating m^6^A marks and leveraging selective recognition by distinct reader proteins, regulate the expression of thermogenesis-related genes, thereby influencing BAT thermogenesis and WAT browning.

In summary, m^6^A modification acts as a precise molecular regulator in thermogenesis control. The balance between FTO and METTL3/METTL14, along with selective recognition by downstream reader proteins, collectively determines the energy metabolic fate of AT (As shown in Figure 3). This complex, context-dependent regulatory network offers numerous potential targets for interventions aimed at increasing energy expenditure and treating obesity.

### 2.7. Regulation of Lipolysis by m^6^A Modification

**Figure 4:**
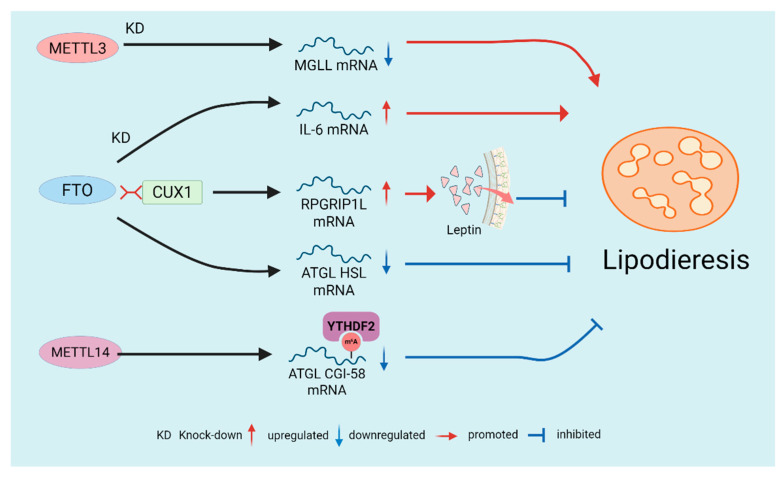
Regulation of Lipolysis by m^6^A Modification. The demethylase FTO generally suppresses lipolysis by influencing multiple downstream targets, including ANGPTL4, IL-6, leptin signaling, and lipolytic enzyme genes. In contrast, the writer enzymes (METTL3/METTL14) typically promote lipolysis by modulating m^6^A modifications and subsequently regulating lipolysis-related genes.

Lipolysis is the metabolic process of breaking down triglycerides (TG) stored in cellular lipid droplets. It is catalyzed by various lipases, including ATGL and hormone-sensitive lipase (HSL), which hydrolyze TG into free fatty acids and glycerol, resulting in reduced lipid droplet size [89]. Multiple studies have shown that m^6^A modification, through the coordinated action of writer and eraser enzymes with reader proteins, influences the breakdown of stored adipose, affecting both intracellular and extracellular lipid degradation(As shown in Figure 4). This highlights the role of m^6^A in energy mobilization and its potential as a target for managing lipid accumulation. Angiopoietin-like proteins 3, 4, and 8 (ANGPTL3, 4, 8) all contribute to triglyceride metabolism [90]. Notably, ANGPTL4 inhibits lipoprotein lipase, thereby suppressing extracellular lipolysis [91]. Studies show that injecting adenovirus encoding ANGPTL4 into adipose-specific *FTO* KO mice restores serum triglyceride levels and lipolytic capacity to levels comparable to controls [92]. This suggests that *FTO* KO affects ANGPTL4 levels in adipocytes and intracellular lipolysis. Furthermore, *FTO* KO increases IL-6 levels in 3T3-L1 cells, promoting the expression of lipolysis-related genes [93]. Research indicates that interactions at binding sites in FTO intron 1 with the transcription factor CUX1 upregulate RPGRIP1L, inhibiting leptin receptor transport and signaling, which reduces lipolysis [94]. Additionally, FTO reduces lipolysis by decreasing the expression of *ATGL* and *HSL* mRNA in AT [95]. Under intermittent hypoxia, downregulation of *METTL3* alters m^6^A modifications and expression of *MGLL* mRNA, promoting lipolysis and demonstrating the context-dependent regulation of lipolysis by m^6^A [96]. Adipose-specific KO of *METTL14* reduces m^6^A modifications, suppressing β-adrenergic signaling and lipolytic gene expression, which promotes obesity and liver lipid accumulation [15]. Recent studies reveal the underlying mechanism: YTHDF2 binds to m^6^A-modified transcripts, inhibiting the synthesis of key lipolytic factors such as ATGL and CGI-58, thereby suppressing lipolysis [97].

### 2.8. Interactions Between m^6^A Modification and Adipokines

AT is also a vital endocrine organ that secretes adipokines in the form of hormones or cytokines, influencing metabolism, inflammation, and immune function locally and systemically. In obesity, AT dysfunction alters its endocrine profile, a key pathophysiological mechanism underlying obesity-related metabolic syndrome. Emerging evidence indicates that m^6^A and adipokines interact through mutual regulation, forming a complex feedback loop that influences systemic metabolism and inflammation (Table 1). This implies that interventions targeting m^6^A could broadly affect endocrine signaling and immune responses related to lipid metabolism. Adiponectin (APN) is an endogenous protein secreted by WAT that enhances energy utilization and insulin sensitivity while exhibiting anti-inflammatory properties [98]. AdipoAI, identified by Yu et al., is an orally active adiponectin receptor agonist that mimics APN’s anti-inflammatory effects [99]. Their study suggests that lncRNAs may participate in AdipoAI’s anti-inflammatory process in LPS-induced macrophages via competing endogenous RNA (ceRNA) networks and m^6^A epigenetic regulation, indicating a potential link between m^6^A modification and APN’s anti-inflammatory actions. Leptin is a hormone secreted by adipocytes that crosses the blood-brain barrier and regulates appetite and energy expenditure through the hypothalamus [100]. Studies report that FTO mediates leptin resistance in HFD-induced obesity. The mechanism involves enhanced FTO/CX3CL1 pathway activity, which upregulates hypothalamic *SOCS3* expression, impairing leptin signal transduction and the STAT3 pathway, leading to leptin resistance and obesity [101]. Additionally, research shows that inhibiting *FTO* in HFD-fed mice increases leptin levels in epididymal white adipose tissue (eWAT), along with elevated inflammatory markers (MCP1, TNF-α), impacting lipid metabolism [102]. Plin5 is a scaffold protein important for lipid droplet formation. Wei et al. found that leptin regulates Plin5 m^6^A methylation by promoting *FTO* expression, affecting lipid metabolism and energy expenditure [78]. Resveratrol (RSV) exerts multiple anti-inflammatory effects. Supplementation with RSV has been reported to ameliorate HFD-induced disturbances in lipid metabolism, an effect that may be associated with reduced m^6^A RNA methylation and increased *PPARα* mRNA expression [103]. The adipokine resistin is closely associated with hepatic steatosis and other fatty liver diseases. Research demonstrates that melatonin in adipocytes enhances m^6^A RNA demethylation to promote resistin mRNA degradation, thereby alleviating endoplasmic reticulum stress-mediated liver steatosis [104]. Retinol-binding protein 4 (RBP4) functions not only as a vitamin A transporter but also as an adipokine that contributes to and exacerbates disturbances in glucose and lipid homeostasis [105]. Recent studies have linked *RBP4* expression and stability to alterations in m^6^A [106]. These findings suggest that m^6^A modification may affect lipid metabolism by regulating RBP4 levels, although the precise mechanisms remain to be elucidated. IL-6 and TNF-α are major pro-inflammatory adipokines. Numerous studies have shown that the levels of m^6^A regulators—writers, erasers, and readers—change in response to TNF-α stimulation. Conversely, TNF-α concentrations are modulated by alterations in m^6^A regulator expression, indicating bidirectional crosstalk between TNF-α and the m^6^A machinery that together shape disease progression [107]. Moreover, perturbations in METTL3 or METTL14 alter the mRNA stability and translation of inflammatory cytokines such as *IL-6* and *TNF-α*, thereby either amplifying or attenuating inflammatory signaling [108,109].

In summary, m^6^A modifications and adipokines form a tightly integrated “Adipo–m^6^A axis.” Adipokines (e.g., leptin, TNF-α, RBP4) can modulate m^6^A writers and erasers in a tissue and context-dependent manner, while m^6^A reciprocally regulates the protein expression and function of these factors by altering their mRNA stability, splicing, nucleo-cytoplasmic transport, and translation. Certain small molecules (for example, resveratrol, melatonin, or adiponectin receptor agonists) may tilt the m^6^A balance to produce multi-target effects and potentially reprogram this network.

## 3. m^6^A Modification in the Regulation of Metabolic Diseases

Dysregulation of the m^6^A modification network serves as a shared pathophysiological basis for various metabolic diseases, linking local lipid metabolism disruptions to systemic metabolic dysfunction. In humans, FTO expression levels correlate with higher serum leptin and lower high-density lipoprotein levels in obese individuals, highlighting the close relationship between m^6^A RNA methylation and glucose-lipid metabolism [110]. Notably, overall m^6^A levels often decrease in tissues from obese and non-alcoholic fatty liver disease (NAFLD) patients, indicating that m^6^A dysregulation is a key feature of these disease states [111].

### 3.1. Role of m^6^A in Hepatic Lipid Homeostasis and Fatty Liver-Related Diseases

**Figure 5:**
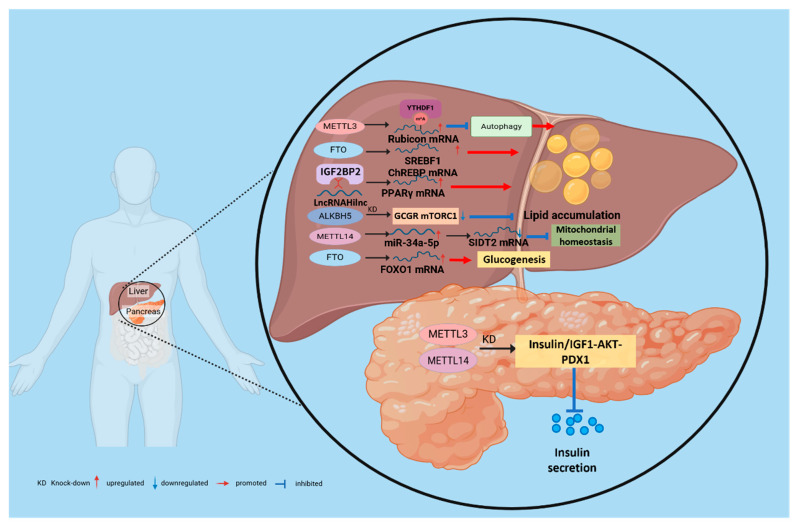
m^6^A-mediated regulatory mechanisms of metabolic diseases in the liver and pancreas. m^6^A “writers,” “erasers,” and “readers” dynamically regulate methylation, thereby modulating the degradation and translation of downstream effectors and controlling pathways such as mitochondrial homeostasis, autophagy, lipid accumulation, and inflammation that together determine hepatic metabolic balance. Furthermore, m^6^A influences pancreatic β-cell proliferation and insulin secretory capacity, thereby affecting insulin resistance and the progression of T2D.

Given the liver’s role as the central organ of systemic energy metabolism, the dysregulation of its m^6^A epitranscriptome has been established as a key factor in the pathogenesis of hepatic steatosis. Multiple studies have revealed multilayered effects of m^6^A on hepatocellular lipid metabolism via distinct molecular pathways: The demethylase FTO reduces global m^6^A levels in hepatocytes, decreases mitochondrial content, and promotes lipid accumulation; FTO also decreases m^6^A methylation on *SREBF1* and *ChREBP* mRNAs, stabilizing these transcripts of key lipogenic transcription factors and thereby promoting steatosis [112,113]. Conversely, m^6^A “writers” also play critical roles in NAFLD: overall m^6^A levels are increased in NAFLD *mouse* models and in hepatocytes treated with free fatty acids, METTL3 directly methylates *Rubicon* mRNA, and YTHDF1 binds the m^6^A-marked transcript to enhance its stability; this suppresses autophagy and leads to lipid-droplet accumulation [114]. Notably, METTL3 functions in a cell-type-dependent manner: hepatocyte-specific *METTL3* KO reduces overall m^6^A levels and produces NAFLD-like pathology [115], whereas myeloid-specific *METTL3* deletion protects against age-related and diet-induced obesity and NAFLD [116]. This stark contrast highlights that the biological outcomes of m^6^A modulation are strictly determined by the cellular context. To provide a clear overview of these complexities, we have summarized the consensus and cell-type-specific roles of major m^6^A regulators in Table 2. Beyond METTL3 and FTO, other m^6^A regulators contribute to pathology through diverse pathways: METTL14 disrupts mitochondrial homeostasis by modulating the miR-34a-5p/SIDT2 axis [117]; Hepatocyte-specific ALKBH5 deletion reduces glucose and lipid levels by inhibiting GCGR and mTORC1 signaling; targeted downregulation of ALKBH5 reverses T2DM and MAFLD phenotypes in diabetic mice, suggesting therapeutic potential [118]. In addition, noncoding RNAs participate in m^6^A-mediated networks: the lncRNA Hilnc interacts with IGF2BP2 to regulate *PPARγ* mRNA stability, thereby contributing to hepatic steatosis [119]. At the signaling level, METTL3 increases m^6^A methylation of *DDIT4* mRNA, thereby modulating mTORC1 and NF-κB pathways. *METTL3* KD reduces lipid accumulation and inflammatory activity in hepatocytes from NAFLD patients [116], indicating that m^6^A regulates the fate of energy-metabolism transcripts and, via autophagy, mitochondrial function, and inflammatory–stress pathways, collectively controls hepatic lipid homeostasis (Figure 5).

### 3.2. Role of m^6^A in Insulin Resistance and Type 2 Diabetes

Insulin resistance, a hallmark of T2D, arises from an impaired response to insulin in peripheral tissues such as the liver and skeletal muscle. Ectopic lipid accumulation in these tissues, a process driven by m^6^A modifications, is a direct cause of insulin resistance. For example, the overexpression of *FTO* in skeletal muscle enhances adipogenesis and oxidative stress, thereby impairing the insulin signaling pathway [120]. Secondly, m^6^A directly regulates hepatic gluconeogenesis. FTO drives excessive glucose production in the liver by demethylating and thereby upregulating the expression of the transcription factor *FOXO1*, which is a key mechanism underlying fasting hyperglycemia [121]. m^6^A modification also plays a decisive role in pancreatic β-cell development, differentiation, and functional maintenance. Loss of *METTL3* disrupts cell fate decisions in pancreatic bipotent progenitors, impairing their differentiation into endocrine or ductal lineages and leading to abnormal islet architecture [122]. Acute deletion of *METTL14* induces endoplasmic reticulum stress by activating the IRE1α/sXBP1 signaling pathway, resulting in impaired glucose tolerance [123]. In addition, IGF2BP2 recognizes and binds m^6^A-modified transcripts, such as *PDX1*, enhancing their stability and translation to sustain insulin secretion in β cells. Conversely, loss or dysfunction of *IGF2BP2* reduces *PDX1* expression, limits β-cell proliferation, and compromises insulin secretory capacity (Figure 5). Consistently, pancreatic islets from patients with T2D exhibit globally reduced m^6^A levels, which closely correlate with β-cell dysfunction and failure [124,125].

### 3.3. m^6^A-Mediated Inter-Organ Communication

**Figure 6:**
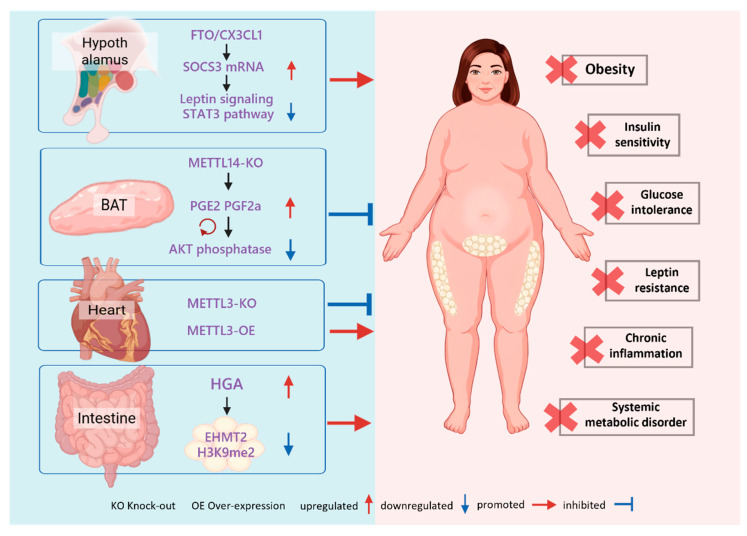
m^6^A-Mediated Systemic Regulation. m^6^A modification serves as a systemic bridge by controlling organ-specific secretomes. Hypothalamic FTO drives obesity through the SOCS3-STAT3 axis, whereas BAT-specific METTL14 deficiency improves insulin sensitivity by boosting prostaglandin secretion. In the heart, METTL3 levels positively correlate with diet-induced metabolic disturbances. Additionally, gut-derived HGA signals to white adipose tissue to induce metabolic dysregulation via m^6^A modulation.

Traditional models of metabolic disease have often focused on cell-autonomous defects within a single organ. However, maintaining metabolic homeostasis in an organism is highly dependent on a complex communication network between different organs. Emerging research reveals a revolutionary concept: m^6^A modification acts as a key mechanism governing this inter-organ dialogue. By controlling the expression of signaling molecules secreted by specific organs, m^6^A modification translates the epitranscriptomic state of one organ into systemic signals that influence whole-body metabolism(As shown in Figure 6). This perspective reflects the multifactorial nature of metabolic diseases and provides a more comprehensive framework for precision medicine. This paradigm shift enables a deeper understanding of how metabolic disorders propagate across tissues and supports the development of interventions that target upstream regulatory signals.

#### 3.3.1. Beyond Cell Autonomy

The consequences of dysregulated m^6^A in a specific tissue extend far beyond the tissue itself. For instance, AT dysfunction is known to increase the release of free fatty acids and pro-inflammatory cytokines, which directly triggers insulin resistance in the liver and muscles—a classic example of pathological inter-organ communication [93,126]. However, recent studies suggest that the regulatory role of m^6^A is more proactive and profound. This modification can directly encode and dispatch regulatory signals, effectively transforming tissues like adipose into endocrine signaling hubs governed by the epitranscriptome.

#### 3.3.2. BAT-Systemic Axis

Studies have found that the adipose-specific KO of *METTL14* in BAT significantly improves systemic insulin sensitivity. Notably, this effect is independent of the classical thermogenic function mediated by UCP1. The underlying molecular mechanism involves the METTL14-mediated m^6^A modification of mRNAs encoding prostaglandin synthases (*PTGES2* and *CBR1*). This methylation marks the transcripts for degradation facilitated by the reader proteins YTHDF2/3. Consequently, when *METTL14* is knocked out, these mRNAs are stabilized, leading to increased synthesis and secretion of prostaglandins PGE2 and PGF2α from BAT. These prostaglandins then enter the circulation and act on distal tissues, where they enhance insulin signaling by inhibiting an AKT phosphatase, thereby improving systemic glucose homeostasis [127,128]. This discovery demonstrates that the epitranscriptomic state of BAT can directly regulate whole-body metabolism through the secretion of lipid signaling molecules.

#### 3.3.3. Heart as an m^6^A-Regulated Endocrine Organ

Compelling evidence also emerges from studies on the heart. Mice with a cardiomyocyte-specific KO of *METTL3* were found to be resistant to weight gain, obesity, and glucose intolerance induced by a Western diet. Conversely, overexpressing *METTL3* in the myocardium exacerbated these metabolic disorders. Studies indicate that m^6^A methylation critically regulates *FGF1*, and that cardiac expression of *FGF1* may have important effects on whole-body metabolism [129]. These findings reveal that the heart can secrete m^6^A-regulated factors that modulate systemic energy balance and adaptive responses to nutritional challenges. Consequently, the heart should be regarded as an active endocrine node within the metabolic regulatory network. Although cardiac factors such as ANP/BNP, FGF16, and YBX1 have been implicated in cardiac disease, the full repertoire of heart-derived metabolic signals remains incompletely characterized [130,131,132]. Future studies combining cardiomyocyte-specific KO models with secretome/proteomic profiling could identify additional m^6^A-regulated secreted mediators and better define the heart’s endocrine potential.

#### 3.3.4. Gut–Microbiota–Adipose Tissue Axis as a Potential Regulatory Pathway

Interactions between the host epitranscriptome and the gut microbiota represent a complex and critical dimension of metabolic regulation. Recent work has reshaped our view of the gut–liver and gut–adipose axes, revealing the microbiota as a powerful regulator of the host m^6^A methylation landscape [133]. Evidence indicates that the gut microbiome is essential for maintaining normal m^6^A topology in the mouse cecum and liver. In germ-free mice, loss of microbial signals causes marked rearrangements of m^6^A patterns, which impair the translation efficiency of transcripts involved in lipid metabolism, inflammation, and circadian regulation [134].These findings define a novel “microbiota–m^6^A–lipid metabolism” axis: dysbiosis perturbs lipid homeostasis not only via microbial metabolites but also by ‘hijacking’ the host epitranscriptomic machinery and rewriting metabolic programs at the level of post-transcriptional modification.

At the molecular level, this communication is primarily mediated by bioactive molecules derived from the microbiota. Diet-induced dysbiosis, for example, following a HFD, results in the release of specific bacterial metabolites (such as homogentisic acid, HGA) and cell-wall components (e.g., lipopolysaccharide, LPS) into the circulation, where they act as distal signaling molecules. These microbial signals target distal organs such as AT, provoking inflammation and directly reshaping the host m^6^A methylation landscape [135,136,137]. For example, exogenous HGA has been shown to increase m^6^A modification of the methyltransferase EHMT2 in eWAT, reducing EHMT2 protein abundance and downstream H3K9me2 levels, thereby precipitating metabolic dysfunction [135]. This mechanism delineates a novel pathological axis that links external environmental triggers (diet), intermediate mediators (microbiota and microbial metabolites such as HGA), and internal regulatory circuits (m^6^A-mediated epitranscriptomic cascades). Recent studies further demonstrate the pathogenic role of this axis in metabolic disease: distinct microbial signatures can differentially modulate the activity of m^6^A writers or erasers, alter the stability of mRNAs encoding key adipogenic and lipolytic genes, and thereby drive obesity and metabolic dysfunction [138,139]. Together, these observations suggest that the gut microbiota functions as an external “writer” of the host metabolic code, and that its perturbation can directly misprogram host metabolic programs.

Based on these findings, interventions targeting this axis hold substantial clinical promise. Chen et al. provide evidence that probiotic interventions or other microbiome-targeted therapies can restore host m^6^A homeostasis and thereby ameliorate lipid metabolic disturbances [140]. These observations are reshaping the etiological framework for metabolic diseases, suggesting that systemic disorders such as T2D or MASLD may not always originate from intrinsic defects in symptomatic organs (e.g., pancreas or liver), but can instead begin with m^6^A dysregulation in the gut—a “signaling organ” that triggers distal cascade effects. Accordingly, future therapeutic strategies should shift from solely treating insulin resistance or hepatic steatosis toward developing agents that precisely target m^6^A pathways in the gut as a signaling organ. This organ-centric, epitranscriptomic communication paradigm aims to correct aberrant inter-organ signaling and thereby open promising new avenues for precision therapies against obesity and metabolic-associated liver disease.

## 4. Therapeutic Strategies Targeting the m^6^A Pathway

Given the central regulatory role of m^6^A in obesity and metabolic diseases, targeting its pathway has become a novel and highly attractive area for drug development. Current research primarily focuses on creating small-molecule inhibitors of m^6^A writer and eraser enzymes (Table 3). However, these approaches differ conceptually: while inhibiting writers or erasers directly alters m^6^A abundance and causes broad, global effects—an approach suited to diseases driven by a single dominant mechanism—targeting readers enables more selective, transcript-specific ‘fine-tuning’ with substantial therapeutic potential but greater development challenges.

### 4.1. Development of FTO Inhibitors for m^6^A Modulation

As the m^6^A modulator with the strongest genetic association to obesity, FTO is the most extensively studied drug target in this field. Significant progress has been made in the development of FTO inhibitors. Several natural compounds have been identified as having FTO-inhibitory activity, including epigallocatechin gallate (EGCG) from green tea and rhein from the traditional Chinese medicine rhubarb [141,146]. While they have shown potential to inhibit adipogenesis and improve metabolism in preclinical studies, these compounds generally suffer from insufficient potency and selectivity. Through drug repositioning and structure-based design, several existing drugs have been found to inhibit FTO. Examples include entacapone, used for Parkinson’s disease, and meclofenamic acid, a non-steroidal anti-inflammatory drug [121,142]. These repurposed drugs offer the potential to accelerate the transition to clinical studies. Concurrently, a series of highly selective small molecules developed through crystal structure analysis and rational design (e.g., FB23, FB23-2, N-CDPCB, CHTB) have demonstrated controllable inhibitory activity and pharmacological effects in vitro and in in vivo oncology models [143]. These compounds have laid the chemical foundation for more potent and selective FTO inhibitors. It is important to note, however, that in vivo validation of these candidates in the context of metabolic diseases is still very limited, with most studies focusing on cancer or proof-of-concept applications [147,148].

### 4.2. Development of METTL3 Inhibitors for m^6^A Modulation

Although the development of METTL3 inhibitors has been predominantly focused on oncology, their potential application in metabolic diseases is beginning to gain attention. STM2457 has been reported as a highly potent and selective small-molecule inhibitor of METTL3 [149]. In *mouse* models of HFD-induced obesity and MASLD, treatment with STM2457 significantly reduced hepatic lipid deposition, decreased body weight, improved glucose tolerance and insulin sensitivity, and lowered liver transaminases and triglyceride levels. These results indicate that pharmacological inhibition of METTL3 can reverse lipid metabolism disorders under certain experimental conditions [144]. Furthermore, the natural product quercetin has also been identified as having METTL3-inhibitory activity [145]. However, applying METTL3 inhibitors to metabolic diseases presents unique challenges. As previously discussed, METTL3 can exert opposing effects in different cell types (e.g., hepatocytes versus myeloid cells). Consequently, systemic inhibition of METTL3 could produce complex or even detrimental net effects. Future therapeutic strategies will likely require the development of cell-type-specific delivery systems to achieve precise and beneficial interventions.

### 4.3. Exploring m^6^A Reader Protein Modulation

Compared to inhibitors of writer or eraser enzymes, which alter global m^6^A levels, targeting reader proteins offers a more nuanced intervention strategy. By inhibiting or activating a specific reader protein, it is theoretically possible to influence only the subset of RNAs bound by that reader. This approach could lead to more precise therapeutic effects with a reduced risk of off-target complications [150]. However, the development of potent and specific small-molecule modulators for m^6^A reader proteins is still in its infancy. This represents a significant knowledge gap and a critical future direction for drug discovery in this field.

### 4.4. Challenges and Future Directions for m^6^A-Targeted Therapies

Despite its promise, translating m^6^A-targeted therapies from bench to bedside faces multiple challenges that must be addressed through precision strategies and emerging technologies to achieve clinically translatable interventions: (1) Long-term safety: Because m^6^A is a fundamental layer of RNA regulation, chronic or global modulation may perturb tumor suppression, neurodevelopment, immune function, and cardiovascular homeostasis, potentially increasing risks such as cancer progression, enhanced inflammation, or stem-cell dysfunction [151,152,153]. These concerns mandate a shift toward precision targeting and comprehensive safety assessment, including long-term animal studies and biomarker monitoring. (2) Specificity: Achieving high selectivity for target enzymes (e.g., FTO, ALKBH5) or individual reader proteins is essential to minimize off-target effects. Emerging tools based on CRISPR–Cas13 enable site- or transcript-specific installation or removal of m^6^A, providing powerful approaches for target validation and specificity optimization [154,155]. Cas13-based systems have demonstrated in vivo RNA-regulatory activity in animal models of antiviral defense and inflammation, offering proof-of-concept for functional validation [156]. Such programmable systems can help bridge the gap between global intervention risks and precision therapeutics by enabling specificity optimization. (3) Tissue and cell-type targeting: Because m^6^A regulators have divergent roles across tissues and cell types, strategies that deliver therapeutics precisely to target tissues (e.g., adipose depots or hepatic macrophages) are essential and represent a key means to reduce risks associated with global m^6^A modulation. Current delivery platforms—including lipid nanoparticles, membrane-modified or cell membrane–coated nanoparticles, exosome–liposome hybrids, and other biomimetic nanoparticles—provide promising frameworks for m^6^A-targeted interventions [157,158,159]. However, these delivery systems must be co-designed with specific m^6^A targets and regulatory strategies, and their tissue specificity, safety, and controllability should be rigorously evaluated in preclinical studies to advance m^6^A-targeted therapies toward precise and translatable clinical applications.

## 5. Conclusions and Future Perspectives

This review describes m^6^A RNA modification as a central component of the epitranscriptome and highlights its pivotal role in regulating lipid metabolism and related metabolic diseases. From controlling adipocyte differentiation and function to mediating complex inter-organ communication and driving the pathophysiology of systemic metabolic disorders (Figure 6), the m^6^A regulatory network exerts critical control over metabolic homeostasis.

Despite rapid progress over the past decade, several crucial regions in the landscape of m^6^A remain to be explored: (1) Cell-Type-Specific m^6^A Profiles and Functions: Current studies predominantly rely on whole tissues or homogenized cell lines. There is an urgent need to utilize high-resolution technologies such as single-cell sequencing to map cell-type-specific m^6^A methylation patterns (methylomes) and their dynamic changes across various metabolic tissues (e.g., adipose, liver, pancreas) and cell types (e.g., adipocytes, hepatocytes, macrophages, endothelial cells, β-cells) in both healthy and diseased human states. This will help explain why the same m^6^A regulator produces markedly different effects in different cells. (2) Molecular Messengers in Inter-Organ Communication: Beyond the identified BAT-prostaglandin axis, the identities of other m^6^A-regulated organokines, their secretion mechanisms, and their modes of action in distal target organs remain a significant “black box.” Elucidating these pathways is a key priority for future research. (3) Crosstalk with Other Epigenetic Modifications: The m^6^A modification does not exist in isolation. How it interacts—either synergistically or antagonistically—with DNA methylation, histone modifications, and other RNA modifications (e.g., m^5^C, m^1^A) to form a higher-order regulatory network for gene expression is still largely unknown. (4) Translation from Animal Models to Humans: The vast majority of current findings are derived from mouse models. The extent to which these discoveries can be translated to human physiology and pathology requires rigorous validation through more extensive human cohort studies and analysis of clinical samples.

In conclusion, a deeper understanding of the m^6^A epitranscriptome not only offers new perspectives and targets for lipid metabolism research but also provides a novel key to unraveling the complex pathogenesis of obesity and metabolic diseases. Precisely modulating this dynamic regulatory network may enable new diagnostic and therapeutic approaches for obesity, T2D, and fatty liver disease and will be instrumental in advancing our understanding and treatment of metabolic syndrome.

## Figures and Tables

**Table 1:** Interactions between m^6^A Modification and Major Adipokines, with Potential Therapeutic Implications.

Adipokine	Primary Source/Tissue	m^6^A-Related Mechanism	Observed Effects/Downstream Signals	Potential Therapeutic Implication	References
APN	WAT	lncRNAs interact with m^6^A-dependent epitranscriptomic mechanisms via ceRNA networks to modulate APN-mediated anti-inflammatory signaling.	m^6^A-related regulation may enhance or mediate the capacity of adiponectin—or adiponectin receptor agonists—to suppress inflammatory responses.	Targeting m^6^A modifications or associated lncRNAs could therefore potentiate the anti-inflammatory effects of APN-based therapies.	[99]
Leptin	Adipocytes secrete factors that act on the hypothalamus.	FTO contributes to HFD-induced leptin resistance. Upregulation of the FTO–CX3CL1 pathway increases hypothalamic SOCS3, which impairs leptin–STAT3 signaling.	FTO-mediated alterations in m^6^A are closely linked to leptin sensitivity, leptin resistance, and the regulation of energy metabolism.	Modulating FTO or m^6^A status may improve leptin responsiveness or modify energy expenditure. Natural interventions—such as RSV—have been associated with changes in m^6^A levels, suggesting peripheral treatments can influence the leptin axis.	[101,103]
		*FTO* inhibition increases leptin levels in eWAT and elevates inflammatory markers, indicating a role for FTO in AT metabolism.	Targeting FTO can therefore alter tissue leptin concentrations and inflammatory biomarker profiles.		[102]
		Leptin regulates the m^6^A methylation status of Plin5 by upregulating *FTO* expression, thereby influencing lipid-droplet formation and lipid metabolism.	Changes in the m^6^A status of Plin5 alter lipid-droplet dynamics and cellular energy metabolism.	These observations indicate that adipokines are not only regulated by m^6^A but can also act upstream to modulate m^6^A-related enzymes, forming feedback regulatory loops.	[78]
Resistin	Adipocyte-secreted factors are implicated in hepatic steatosis.	Melatonin promotes m^6^A demethylation of resistin mRNA, facilitating its degradation and reducing intracellular resistin levels.	m^6^A demethylation decreases resistin mRNA stability, which may alleviate endoplasmic reticulum (ER) stress–associated hepatic steatosis.	Promoting resistin mRNA degradation—for example, via melatonin treatment or modulation of relevant m^6^A demethylases—could represent a potential strategy to improve hepatic steatosis.	[104]
ANGPTL4	Adipocytes/liver	FTO status influences cellular ANGPTL4 levels; ANGPTL4 inhibits LPL and extracellular TG hydrolysis	Modulates extracellular lipolysis via LPL inhibition; impacts circulating TG and lipolytic capacity	Targeting the FTO–ANGPTL4 axis could restore lipolysis and normalize circulating triglycerides	[92]
IL-6	Adipocytes, AT macrophages	m^6^A influences *IL-6* mRNA stability/translation; *FTO* KO elevates IL-6 in adipocyte models.	Increased IL-6 promotes inflammatory signaling and can stimulate lipolytic.	m^6^A-directed interventions could modulate inflammatory tone and lipolysis in AT.	[93,108]
TNF-α	AT macrophages/adipocytes	m^6^A regulation can alter TNF-α transcript stability and expression (context-dependent)	Drives local and systemic inflammation, contributes to insulin resistance	m^6^A targeting might reduce chronic adipose inflammation and improve insulin sensitivity	[107]

**Table 2:** Summary of Cell-Type-Specific Roles of m^6^A Regulators.

Regulator	Tissue/Cell Type	Condition/Context	Biological Outcome (Effect on Lipid Metabolism)	Molecular Mechanism/Target	Consensus or Context-Dependent	References
FTO	Preadipocytes	Adipogenesis	Promotes (Differentiation)	Splicing of RUNX1T1; Cell cycle regulation (CCNA2)	Consensus (Generally pro-adipogenic)	[52,54]
FTO	Hepatocytes	Lipogenesis/MASLD	Promotes (Steatosis)	Demethylates SREBF1, ChREBP (Stabilizes lipogenic factors)	Consensus	[112]
METTL3	Preadipocytes (BMSCs)	Adipogenesis	Inhibits (Differentiation)	Methylates JAK1 (Decay), FOXO1 (Translation)	Dominant View (But complex targets)	[58,59]
METTL3	Hepatocytes	MASLD/Lipotoxicity	Protective (Prevents Steatosis)	Maintains circadian/metabolic homeostasis (KO leads to MASLD)	Cell-Type Dependent (Contrasts with myeloid role)	[114]
METTL3	Hepatocytes	HFD/FFA	Promotes (Lipid Accumulation)	Methylates Rubicon (Suppresses Autophagy)	Context-Dependent (Specific to autophagy inhibition)	[113]
METTL3	Myeloid Cells (Macrophages)	Inflammation/MASLD	Promotes (Pathology)	Methylates inflammatory cytokines; Activates Macrophages	Cell-Type Specific (Opposite to hepatocyte function)	[115]
METTL3	BAT	Thermogenesis	Promotes (Energy Expenditure)	Stabilizes PRDM16, UCP1	Consensus (Essential for BAT identity)	[80]

**Table 3:** Drugs and Compounds Targeting the m^6^A Pathway for Metabolic Disease Research.

Compound/Drug	Target	Class	Key Effects in Preclinical Metabolic Models	References
Entacapone	FTO	Drug Repositioning	Inhibits adipogenesis, improves insulin sensitivity, and drives hepatic gluconeogenesis via FOXO1.	[121]
EGCG	FTO	Natural Product	Inhibits adipocyte differentiation and increases m^6^A levels.	[141]
Meclofenamic acid	FTO	Drug Repositioning	Inhibits FTO activity and increases energy expenditure.	[142]
FB23	FTO	Small-Molecule Inhibitor	Ameliorates HFD-induced obesity, metabolic dysfunction, and cognitive decline.	[102]
N-CDPCB/CHTB	FTO	Small-Molecule Inhibitor	Increases mRNA m^6^A levels in 3T3-L1cell.	[143]
STM2457	METTL3	Small-Molecule Inhibitor	Reduces hepatic lipid deposition, decreases body weight, and improves glucose tolerance and insulin sensitivity.	[144]
Quercetin	METTL3	Natural Product	Inhibits METTL3 activity and reduces m^6^A levels at the cellular level.	[145]

## Data Availability

No new data were created or analyzed in this study.

## Abbreviations

3′-UTRs	3′ untranslated regions
α-KG	2-oxoglutarate
ALKBH5	AlkB homolog 5
ANGPTL3, 4, 8	Angiopoietin-like proteins 3, 4, and 8
APN	Adiponectin
AT	adipose tissue
BAT	brown adipose tissue
ceRNA	competing endogenous RNA
EGCG	epigallocatechin gallate
eWAT	epididymal white adipose tissue
FASN	fatty acid synthase
FTO	fat mass and obesity-associated
HAKAI	E3 ubiquitin-protein ligase
HFD	high-fat diet
HGA	homogentisic acid
HIF1A	hypoxia-inducible factor 1α
HNRNP	heterogeneous nuclear ribonucleoprotein
HSL	hormone-sensitive lipase
IGF2BP1–3	insulin-like growth factor 2 mRNA-binding proteins 1–3
KD	knockdown
KO	knockout
LPS	lipopolysaccharide
m^1^A	N^1^-methyladenosine
m^6^A	N^6^-methyladenosine
m^6^Am	N^6^,2′-O-dimethyladenosine
MACOM	m^6^A-METTL associated complex
MASLD	metabolic dysfunction-associated steatotic liver disease
MCE	mitotic clonal expansion
METTL3	Methyltransferase-like 3
METTL14	Methyltransferase-like 14
METTL16	Methyltransferase-like 16
MTC	methyltransferase complex
NADP^+^	nicotinamide adenine dinucleotide phosphate
NAFLD	non-alcoholic fatty liver disease
RBP4	Retinol-binding protein 4
RSV	Resveratrol
TG	triglycerides
T2D	type 2 diabetes
UCP1	uncoupling protein 1
VIRMA	Vir-like m^6^A methyltransferase associated protein
WAT	white adipose tissue
WTAP	Wilms’ tumor 1-associating protein
YTH	YT521-B homology
ZC3H13	Zinc finger CCCH domain-containing protein 13
